# Dynamic Changes in EPCAM Expression during Spermatogonial Stem Cell Differentiation in the Mouse Testis

**DOI:** 10.1371/journal.pone.0023663

**Published:** 2011-08-15

**Authors:** Mito Kanatsu-Shinohara, Seiji Takashima, Kei Ishii, Takashi Shinohara

**Affiliations:** 1 Department of Molecular Genetics, Graduate School of Medicine, Kyoto University, Kyoto, Japan; 2 Japan Science and Technology Agency, CREST, Kyoto, Japan; Stanford University, United States of America

## Abstract

**Background:**

Spermatogonial stem cells (SSCs) have the unique ability to undergo self-renewal division. However, these cells are morphologically indistinguishable from committed spermatogonia, which have limited mitotic activity. To establish a system for SSC purification, we analyzed the expression of SSC markers CD9 and epithelial cell adhesion molecule (EPCAM), both of which are also expressed on embryonic stem (ES) cells. We examined the correlation between their expression patterns and SSC activities.

**Methodology and Principal Findings:**

By magnetic cell sorting, we found that EPCAM-selected mouse germ cells have limited clonogenic potential in vitro. Moreover, these cells showed stronger expression of progenitor markers than CD9-selected cells, which are significantly more enriched in SSCs. Fluorescence-activated cell sorting of CD9-selected cells indicated a significantly higher frequency of SSCs among the CD9^+^EPCAM^low/-^ population than among the CD9^+^EPCAM^+^ population. Overexpression of the active form of EPCAM in germline stem (GS) cell cultures did not significantly influence SSC activity, whereas EPCAM suppression by short hairpin RNA compromised GS cell proliferation and increased the concentration of SSCs, as revealed by germ cell transplantation.

**Conclusions/Significance:**

These results show that SSCs are the most concentrated in CD9^+^EPCAM^low/-^ population and also suggest that EPCAM plays an important role in progenitor cell amplification in the mouse spermatogenic system. The establishment of a method to distinguish progenitor spermatogonia from SSCs will be useful for developing an improved purification strategy for SSCs from testis cells.

## Introduction

Spermatogonial stem cells (SSCs) account for a small population of testis cells [1], [2], and their self-renewal activity distinguishes them from committed progenitor cells. Spermatogonia, the most undifferentiated germ cells in testes, contain both SSCs and progenitor cells. SSCs are able to reproduce themselves while producing progenitor cells, thereby maintaining a constant population size. In contrast, progenitor spermatogonia disappear after several rounds of mitotic division. Self-renewal activity is defined only through retrospective analysis of daughter cells, making it difficult or impossible to identify SSCs by morphological observation.

In 1994, a germ cell transplantation technique was developed, in which donor testis cells recolonize seminiferous tubules following microinjection into the testes of infertile recipients [3]. This provided the first functional assay for SSCs. The estimated number of SSCs was 2×10**^3^** to 3×10**^3^** per testis, which represents ∼10% of the total A_single_ (A_s_) spermatogonia, suggesting that only a small population of A_s_ cells have SSC activity [2], [4], [5]. Using the functional transplantation assay, SSCs were subsequently analyzed for the expression of cell surface markers by selecting cells with monoclonal antibodies against surface antigens [6], [7]. Although no SSC-specific markers have been identified, several markers for SSCs are available [8], and a combination of positive and negative selection by surface antigens has allowed the purification of SSCs to 1 in 15 to 30 purified cells [6], [7]. However, the degree of enrichment achieved using individual antigens is limited and ranges from 1∶625 to 1∶1250 [6]–[8], suggesting that committed spermatogonia express similar markers.

In this study, we analyzed the expression of CD9 and epithelial cell adhesion molecule (EPCAM) on SSCs. CD9 is a member of the tetraspanin family molecules and is expressed on mouse and rat SSCs [9]. On the other hand, EPCAM is a homophilic, calcium-independent cell adhesion molecule and is uniquely expressed on the germline cells from the embryonic stages of germ cell development. Its expression in the postnatal testis continues until the spermatocyte stage [10]. Although both of these antigens have been used to purify SSCs, EPCAM was the more useful marker for purifying rat SSCs [11]. However, while attempting to initiate SSC cultures from mouse testes, we observed that EPCAM-expressing cells had limited ability to produce spermatogonial colonies. Flow cytometric analysis revealed that EPCAM expression changed dynamically during spermatogonial differentiation. Here, the identity of EPCAM-expressing cells was determined by germ cell transplantation assay, and the function of EPCAM was analyzed by in vitro spermatogonial culture.

## Materials and Methods

### Ethics statement

We followed the Fundamental Guidelines for Proper Conduct of Animal Experiment and Related Activities in Academic Research Institutions under the jurisdiction of the Ministry of Education, Culture, Sports, Science and Technology, and all of the protocols for animal handling and treatment were reviewed and approved by the Animal Care and Use Committee of Kyoto University (Med Kyo 11079).

### Animals

ICR mice (Japan SLC, Shizuoka, Japan) were used for primary testis cell culture. Transgenic mouse line C57BL/6 Tg14(act-EGFP)OsbY01 (designated as Green; a gift from Dr. M. Okabe, Osaka University, Osaka, Japan) was used for transplantation experiments using magnetic cell sorting (MACS). Transgenic mouse line B6-TgR(ROSA26)26Sor (designated as ROSA; purchased from the Jackson Laboratory, Bar Harbor, ME) was used for fluorescence-activated cell sorting (FACS) experiments to avoid interference of enhanced green fluorescent protein (EGFP) fluorescence for multiparameter sorting. ROSA mice that were backcrossed to the DBA/2 background for more than eight generations were used for derivation of germline stem (GS) cells [12]. WBB6F1-W/W^v^ (W) mice (Japan SLC) were used as recipients for germ cell transplantation.

### Cell culture

For characterization of spermatogonia in the pup testis, testis cells were prepared from 7- to 10-day-old male mice. Single-cell suspensions were obtained by two-step enzymatic digestion using collagenase type IV (1 mg/ml) and trypsin (0.25%), as described previously [12], [13]. Cells were plated at 3×10^5^ cells / well of 6-well culture plates, which have been coated with laminin (20 µg/ml; BD Biosciences, Franklin Lakes, NJ). GS cells were derived from ROSA mice, and were maintained on mitomycin C-treated mouse embryonic fibroblasts (MEFs) [12], [13]. Culture medium was prepared by modifying commercial medium (StemPro^®^-34 serum-free medium (SFM); Invitrogen, Carlsbad, CA) as described previously [12], [13]. Growth factors used were human fibroblast growth factor 2 (FGF2;10 ng/ml) and rat glial cell line-derived neurotrophic factor (GDNF; 15 ng/ml; both from Peprotech, Rocky Hill, NJ).

For overexpression of the intracellular fragment of EPCAM (EpICD) [14], the cDNA fragment encoding EpICD (a gift from Dr. O. Gires, Ludwig Maximilian University of Munich, Germany) was cloned into *CSII-EF-IRES2-Venus* vector. Lentivirus particles were produced by transient transfection of 293T cells, and GS cells from ROSA mice (ROSA GS cells) were transfected, as described previously [15]. For EpICD overexpression experiments, the virus titer was determined by transfecting 293T cells, and the multiplicities of infection (MOI) was adjusted to 2.0. For short hairpin RNA (shRNA)-mediated gene knockdown (KD), the *Epcam* KD vectors TRCN0000111220, TRCN0000111221, TRCN0000111222, TRCN0000111223, and TRCN0000111224 were purchased from Open Biosystems (Huntsville, AL). A mixture of lentivirus particles was used to transfect GS cells from ROSA mice, and 3 independent samples were examined. A lentivirus expressing shRNA against EGFP was used as a control (Open Biosystems). The lentivirus titer was determined using a Lenti-X p24 rapid titer kit (Clontech, Mountain View, CA). The MOI in the KD experiment was adjusted to 24.0.

### Cell separation and flow cytometry

Testis cells were prepared from 5- to 10-week-old male mice. MACS was performed as described previously using rat anti-mouse EPCAM (G8.8; Biolegend, San Diego, CA) or rat anti-mouse CD9 (KMC8; BD Biosciences) antibodies [9], [16]. Sheep anti-rat IgG Dynabeads (Invitrogen) were used for in vitro culture, and goat anti-rat IgG microbeads (Miltenyi Biotech, Gladbach, Germany) were used as a secondary antibody for FACS experiments. The average recovery was determined by four experiments.

For analyses of cell surface antigens, CD9- or EPCAM-selected cells were incubated with the following antibodies: rat anti-mouse CD9 (2B8; BD Biosciences), rat anti-mouse EPCAM (G8.8; Biolegend), mouse anti-mouse FUT4 (SSEA1; MC-480; eBioscience, San Diego, CA), biotin-conjugated anti-mouse ITGB1 (Ha2/5; BD Biosciences), and rat anti-mouse ITGA6 (GoH3; BD Biosciences). Secondary reagents were: allophycocyanin (APC)-conjugated anti-rat IgG, APC-conjugated streptavidin, and APC-conjugated anti-mouse IgM (all from BD Biosciences). For double immunostaining, CD9-selected cells were incubated with APC-conjugated rat ant-CD9 and phycoerythrin (PE)-conjugated anti-EPCAM antibodies. PE-Cy7-conjugated KIT antibody (eBioscience) was used to evaluate KIT expression in subfractionated cells. The cells were incubated in ice-cold phosphate-buffered saline/1% fetal bovine serum (PBS/1% FBS). EpICD-transfected ROSA GS cells were sorted according to Venus expression. Propidium iodide (1 µg/ml; Sigma, St. Louis, MO) was added to exclude dead cells. Stained cells were analyzed by FACSCalibur or sorted by FACSAria II (both from BD Biosciences).

### Apoptosis assay

For terminal deoxynucleotidyl transferase dUTP nick end labeling (TUNEL) staining, single cell suspension was concentrated on glass slides by centrifugation with Cytospin 4 (Thermo Electron Corporation, Cheshire, UK). After fixation in 4% paraformaldehyde for 1 h, cells were then labeled using an In situ Cell Death Detection kit; TMR red (Roche Applied Science, Mannheim, Germany) following the manufacturer's protocol. The cells were couterstained with Hoechst 33342 (2 µg/ml; Sigma) to determine the percentage of TUNEL-positive nuclei relative to the total number of cells. Apoptotic cells were quantified by collecting three images using Photoshop software (Adobe Systems, San Jose, CA). At least 200 cells were counted for each sample.

### Germ cell transplantation

Germ cell transplantation was performed by microinjection into the seminiferous tubules via the efferent duct [17]. Approximately 75–85% of the tubules were filled in each recipient testis. At least three experiments were carried out for MACS and FACS. In experiments using GS cells, recipient mice were treated with anti-CD4 antibody (GK1.5; gift from Dr. T. Honjo, Kyoto University) to avoid rejection of allogeneic donor cells [18]. The Institutional Animal Care and Use Committee of Kyoto University approved all of the animal experiment protocols.

### Analysis of the recipient testes

In experiments using ROSA mice, the recipient testes were fixed with 4% paraformaldehyde for 2 h, and LacZ staining was performed using 5-bromo-4-chloro-3-indolyl ß-D-galactoside (X-Gal) (Wako Pure Chemical Industries, Osaka, Japan), as described previously [5]. In experiments using Green mice, the recipient testes were analyzed under UV light. These methods specifically identify donor cells, because host cells do not stain for LacZ and lack green fluorescence. We defined colonies as donor cell clusters longer than 0.1 mm occupying the entire circumference of the seminiferous tubule. Results were obtained from analyses of 10–12 recipient testes in at least two experiments. For histological analyses, samples were embedded in paraffin blocks and sectioned. The sections were counterstained with hematoxylin and eosin.

### Analysis of gene expression

Total RNA was extracted using Trizol, and first-strand cDNA was synthesized by reverse transcription with Superscript^TM^ II (both from Invitrogen) for reverse transcriptase-polymerase chain reaction (RT-PCR). For quantifying mRNA expression using real-time PCR, a StepOnePlus^TM^ Real-Time PCR system and *Power* SYBR Green PCR Master Mix were used according to the manufacturer's protocol (Applied Biosystems, Warrington, UK). Transcript levels were normalized to that of *Hprt,* with expression levels in EPCAM-selected cells. The PCR conditions were 95°C for 10 min, followed by 40 cycles of 95°C for 15 s and 60°C for 1 min. Each PCR was run at least in triplicate using specific primers (Table S1).

### Statistical analysis

The results were presented as means±SEM. Independent samples with equal variance were analyzed using the Student's *t*-test. SSC activity of subfractionated cells was analyzed by ANOVA followed by Tukey's HSD.

## Results

### Reduced SSC potential of EPCAM^ +^ cells

**Figure 1:**
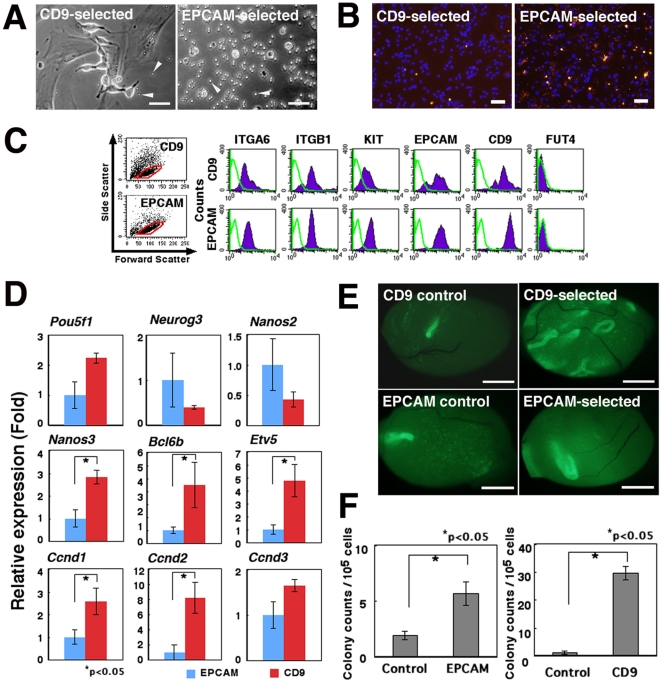
Characteristics of germ cells after CD9 or EPCAM selection. (A) Appearance of CD9-selected (Left) or EPCAM-selected (Right) germ cells on laminin-coated dishes, after 7 days in culture. Testis cells were collected from 10-day-old pups and used to initiate GS cell cultures after MACS. No significant colony formation is seen in EPCAM-selected cells. CD9-selected cells started to proliferate to form spermatogonia chains under the same culture condition, and spermatogonial chains are seen. Note the contaminating testicular fibroblasts in cultures of CD9-selected cells. Arrows indicate magnetic beads used for cell separation. (B) Apoptosis of CD9-selected (Left) and EPCAM-selected cells (Right), after 8 days in culture. TUNEL-positive cells are stained red. Counterstained by Hoechst 33342 (blue). (C) Flow cytometric analyses of CD9-selected (Top) or EPCAM-selected (Bottom) cells collected from adult testes. Green lines indicate control staining. Note the increased KIT staining in EPCAM-selected cells. (D) Real-time PCR analyses of spermatogonial marker genes or cyclins in EPCAM- or CD9-selected cells. CD9-selected cells show increased expression of *Nanos3, Bcl6b, Etv5, Ccnd1*, and *Ccnd2*. Transcript levels were normalized to *Hprt* expression, with expression levels in EPCAM-selected cells. (E) Macroscopic appearance of recipient testes transplanted with EPCAM- or CD9-selected cells. Green tubules indicate germ cell colonies developed from donor SSCs. The same numbers of cells were transplanted at the same time. (F) Quantification of colonies. Both EPCAM-selected (Left) and CD9-selected (Right) cells produced significantly more germ cell colonies than control unselected testis cells, but CD9-selected cells contained a higher concentration of SSCs. Bars  = 20 µm (A); 100 µm (B); 1 mm (E).

Testicular somatic cells often overwhelm growth of proliferating germ cells in vitro [12]. To establish an improved strategy for SSC culture initiation, we assumed that EPCAM would be a useful selection marker because it is thought to be expressed specifically in germ cells, including SSCs [8], [10]. In preliminary experiments, we used anti-EPCAM antibody to collect EPCAM-expressing germ cells from pup testes, which are relatively enriched for SSCs owing to the absence of differentiating germ cells [19]. Testis cells were prepared from 7-day-old pups, and EPCAM-expressing cells were collected by MACS. The cells were cultured on laminin-coated plates. Although CD9-selected cells contained testicular somatic cells that over-proliferated and interfered with germ cell proliferation in culture, the majority of the EPCAM-selected cells consisted of a pure population of germ cells; only a few somatic cells were found (Fig. 1A). However, proliferation of the EPCAM-selected cells was limited, and many of the cells eventually underwent apoptosis, which was identified by TUNEL staining (5.0±0.8% vs. 28.7±0.4%, respectively, for CD9- and EPCAM-selected cells; Fig. 1B). In contrast, cultures initiated with CD9-selected cells exhibited typical spermatogonial proliferation and colony growth by 7–10 days.

As these results indicated that EPCAM-selected cells have limited clonogenic potential in vitro, we characterized the EPCAM- and CD9-selected cells. EPCAM- and CD9-expressing cells were collected by MACS and stained for several cell surface markers known to be expressed on germline cells [8], [20] (Fig. 1C). Although both EPCAM- and CD9-selected cells expressed markers of SSCs, expression of KIT, which is a marker for progenitor spermatogonia [20], was stronger in EPCAM-selected cells, which suggested that they included more progenitor spermatogonia. In addition, CD9-selected cells also contained a significant proportion of cells that did not express EPCAM. Real-time PCR analysis of genes thought to be involved in SSC self-renewal and differentiation revealed reduced expression of *Nanos3*, *Bcl6b*, and *Etv5* in EPCAM-selected cells [8], [21]–[23] (Fig. 1D). The expression levels of *Ccnd1* and *Ccnd2*, which influence SSC activity [24], were also downregulated. These results suggest that EPCAM-selected cells have reduced SSC activity.

To directly test this hypothesis, EPCAM- or CD9-expressing cells were collected and their SSC activities were assessed by germ cell transplantation. The average recoveries of EPCAM- and CD9-selected cells were 4.9±1.0% and 5.9±0.5% of the total testis cells, respectively. The selected cells were microinjected into the seminiferous tubules of congenitally infertile W mouse testes. Two months later, the recipients were killed, and the number of colonies in the testes was analyzed under UV fluorescence (Fig. 1E). EPCAM-selected cells produced significantly more colonies compared with non-selected control cells (5.7±1.0 vs. 1.9±0.4 colonies/10^5^ transplanted cells, respectively; Fig. 1F). In contrast, in two transplantation experiments, the CD9-selected cells resulted in more efficient SSC recovery, producing 29.5±2.3 colonies/10^5^ transplanted cells, which was 32.8 times the number of colonies from the control cells (0.9±0.6 colonies). These experiments suggest that the concentration of SSCs is higher among CD9-selected cells than EPCAM-selected cells.

### Subfractionation of CD9-selected cells by EPCAM expression level

**Figure 2:**
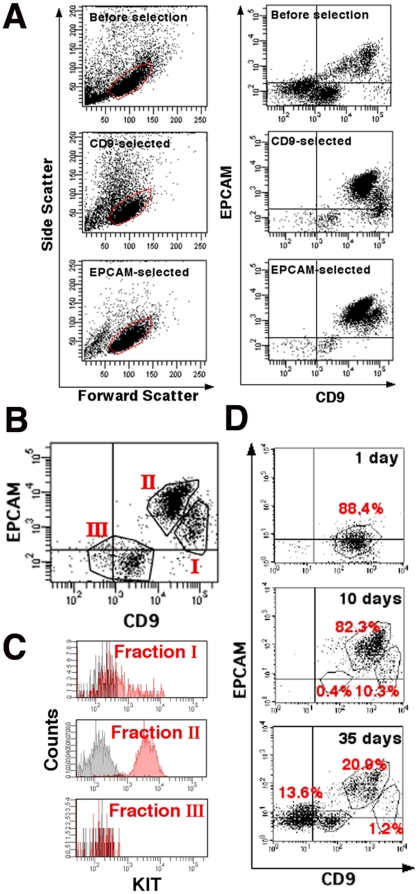
Flow cytometric analyses of CD9- or EPCAM-selected cells after MACS. (A) Light-scattering properties and double immunostaining of total testis cells (Top), CD9-selected cells (Middle) or EPCAM-selected cells (Bottom) stained with APC-conjugated anti-CD9 and PE-conjugated anti-EPCAM antibodies. Cells were gated according to forward scatter (size) and side scatter (cell complexity) values (Left). Gated cells were analyzed for CD9 and EPCAM (Right). Note the simpler light-scattering properties of EPCAM-selected cells. (B) Three subpopulations of CD9-selected cells. Fraction I shows high CD9 and low or no EPCAM immunostaining; fraction II shows low CD9 and high EPCAM immunostaining; and fraction III is low or no CD9 and no EPCAM immunostaining. (C) The three CD9-selected cell subpopulations, immunostained with APC-conjugated anti-CD9, PE-conjugated anti-EPCAM, and PE-Cy7-conjugated anti-KIT antibodies. KIT is strongly expressed in fraction II. Areas shaded in black indicate control staining. (D) Changes in immunostaining of total testis cells during postnatal testicular development. Total testis cells were stained with APC-conjugated anti-CD9 and PE-conjugated anti-EPCAM antibodies. Stronger CD9 and EPCAM immunostaining is seen in 10-day-old mouse testis cells compared with 1-day-old mouse testis cells. Only testes from 35-day-old mice show all three fractions.

To investigate the difference between EPCAM- and CD9-selected cells in the transplantation experiments, we next analyzed the expression of EPCAM and CD9 in EPCAM- and CD9-selected cells of ROSA mice recovered by MACS. Flow cytometry of the double-stained spermatogonial populations was performed by gating according to cell size (forward scatter) and complexity (side scatter). As expected from the result of MACS experiments, CD9-selected cells contained cells with relatively high side scatter values, which suggested their heterogeneity (Fig. 2A). In contrast, EPCAM-selected cells were more uniform in size and complexity. Double-stained CD9-selected cells revealed the presence of at least three subpopulations (Fig. 2B). Fraction I consisted of cells exhibiting strong CD9 expression with relatively weak EPCAM expression. Fraction II, which contained significantly more cells than fraction I, comprised cells with strong EPCAM expression and medium CD9 expression. A CD9^low/-^ population of cells that lacked EPCAM constituted fraction III. Compared with the CD9-selected cells, the EPCAM-selected cells showed a distinct forward scatter/side scatter profile and consisted predominantly of fraction II cells. They also contained only EPCAM^+^ cells of fraction I cells (Fig. 2A). KIT expression was stronger in fraction II than in fraction I cells, suggesting that fraction I cells were more undifferentiated (Fig. 2C). Fraction III showed little KIT expression (Fig. 2C).

We then analyzed testis cells from three different developmental stages to examine when these subpopulations appear during testicular development. We collected testis cells from 1-, 10-, and 35-day-old mice and stained them with CD9 and EPCAM antibodies (Fig. 2D). Testis cells from 1-day-old mice contained only gonocytes, and showed predominantly CD9^+^ cells, with very few EPCAM^+^ cells. EPCAM^+^ cells were found in 10-day-old mouse testis cells, which contained spermatogonia and spermatocytes, but not spermatids. Testis cells at this stage could be separated into two subpopulations, fraction I (CD9^+^EPCAM^low/-^) and fraction II (CD9^low^EPCAM^+^), with fraction I being significantly smaller. Testis cells from 35-day-old mice contained all stages of spermatogenic cells, and revealed three subpopulations; the relative proportion of cells in fraction I was smaller than that in 10-day-old mouse testis cells, possibly reflecting increased production of differentiating meiotic or haploid cells. These results suggest an enrichment of fraction I (CD9^+^EPCAM^low/-^) in spermatogonia.

**Figure 3:**
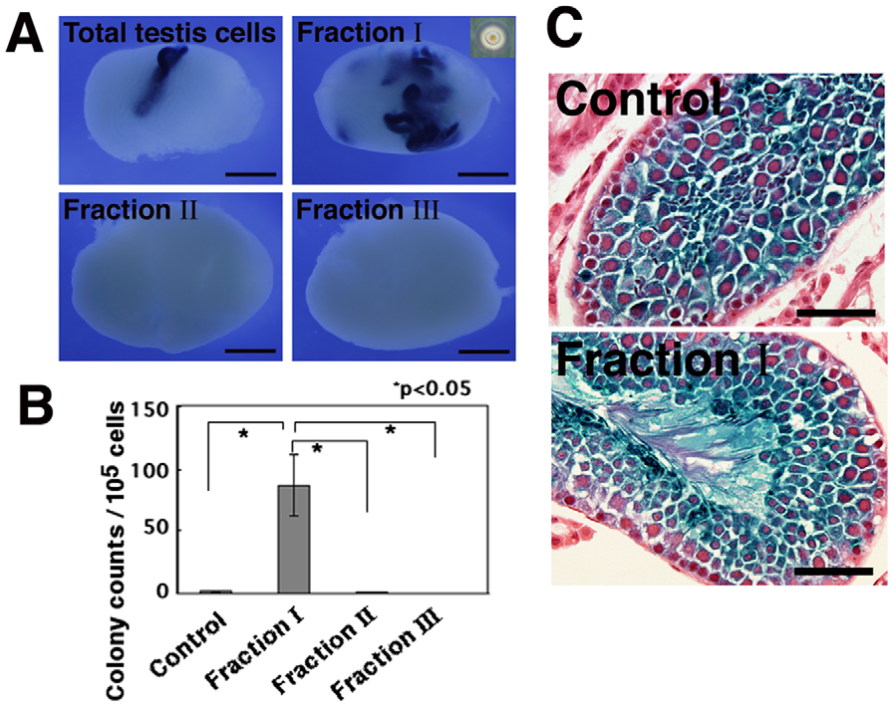
Functional analyses of SSC activity by germ cell transplantation of each CD9-selected cell fraction. (A) Macroscopic appearance of recipient testes. Approximately 4.9×10^3^, 2.3×10^4^, 3.5×10^2^, and 1.6×10^5^ cells were transplanted for fractions I, II, III, and control cells, respectively. Recipient testes were stained with X-Gal 2 months after transplantation. Blue tubules indicate germ cell colonies developed from donor SSCs. Cells in fraction I have a uniform appearance (insert). (B) Quantification of colonies. The number of cells that could be recovered in each experiment varied, and thus the colony number was normalized to reflect donor cells at a concentration of 10^5^ cells injected/testis. Cells in fraction I are significantly enriched for SSCs. (C) Histological sections of recipient testes. Note the normal appearance of spermatogenesis of donor-derived cells in recipients of control cells (Top) and fraction I cells (Bottom). Stain: X-Gal (A); X-Gal, Hematoxylin and eosin (C). Bars  = 1 mm (A), 50 µm (C).

To determine which CD9-selected cell fraction was enriched for SSCs, cells from each of the three fractions were transplanted into the seminiferous tubules of recipient mice. Non-selected total testis cells were used as a control. Fraction I cells exhibited the highest SSC activity in recipient testes, producing 86.6±24.4 colonies/10^5^ transplanted cells (Fig. 3A and B). The concentration of SSCs in this fraction was ∼48.7-fold that in control cells, which produced 1.8±0.5 colonies/10^5^ transplanted cells. Consistent with this result, microscopic analysis of the sorted cells showed that fraction I consisted of cells with a relatively uniform appearance and occasional pseudopod formation (Fig. 3A, inset). Histological sections confirmed the normal appearance of the transplanted cells (Fig. 3C). Although fraction II also contained some SSCs (1.1±0.6 colonies/10^5^ cells transplanted), no significant enrichment was observed compared with non-selected control testis cells. Fraction III cells had no SSC activity.

### Analysis of EPCAM function

**Figure 4:**
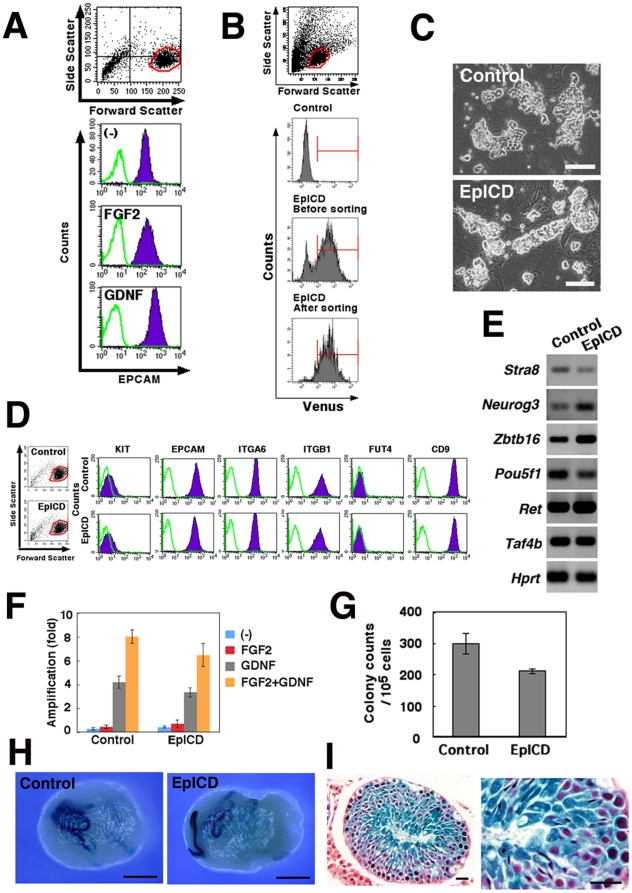
Overexpression of EpICD in ROSA GS cells. (A) Upregulation of EPCAM by GDNF stimulation. ROSA GS cells were cultured on laminin with 1% FBS and without cytokines for 3 days and then stimulated with the indicated cytokines. The cells were recovered 3 days after cytokine stimulation, and stained with anti-EPCAM antibody. (B) Sorting of EpICD-transfected GS cells. GS cells (3×10^5^) on MEFs in 6-well plates were infected, expanded in vitro, and sorted. (C) Morphology of the sorted cells. (D, E) Flow cytometric (D) and RT-PCR (E) analyses of EpICD-transfected ROSA GS cells. No significant changes are seen. Green lines indicate controls. (F) Effects of cytokines on proliferation of EpICD-transfected ROSA GS cells. GS cells (3×10^5^) on MEFs were cultured with the indicated cytokines and recovered by trypsinization 6 days after initiation of culture. No significant differences are seen between the control and EpICD-transfected cells. (G) Quantification of colonies. No significant differences are seen between the control and EpICD-transfected cells. (H) Macroscopic appearance of recipient testes. Recipient testes were stained with X-Gal 2 months after transplantation. Blue tubules indicate germ cell colonies developed from donor SSCs. (I) Histological sections of recipient testes. Cells show apparently normal spermatogenesis. Stain: X-Gal (H); X-Gal, Hematoxylin and eosin (I). Bars  = 100 µm (C), 1 mm (H), 20 µm (I).

To investigate the function of EPCAM, we used the GS cell culture system, in which SSCs increase their numbers exponentially in vitro in the presence of GDNF and FGF2 [12]. GS cells were previously shown to express EPCAM, and 1–2% of GS cells had SSC activity [12], [25]. Flow cytometric analyses showed that the EPCAM expression level in GS cells was upregulated by supplementation with GDNF, whereas FGF2 showed no apparent effect (Fig. 4A).

Considering the expression of EPCAM on embryonic stem (ES) cells and rat SSCs, we examined whether stimulation of EPCAM increases SSC activity. GS cells from ROSA mice were infected with a lentivirus expressing the intracellular domain of EPCAM (EpICD) as well as Venus protein under the control of the *EF-1 α*promoter. Normally in cells, EpICD is normally cleaved after EPCAM activation, and thus the EpICD protein can transmit the signal to the nucleus. *Venus*-expressing cells were purified and cultured in vitro for expansion (Fig. 4B and C). However, the transfected cells did not undergo a significant change in cell or colony morphology (Fig. 4C). In addition, flow cytometric analyses showed no change in the expression level of EPCAM or any other spermatogonial marker examined (Fig. 4D). There were no significant changes in gene expression patterns as determined by RT-PCR or in responses to exogenous cytokines (Fig. 4E and F) [8], [26].

To look at the effect of EPCAM stimulation on SSC self-renewal, we transplanted EpICD-expressing GS cells into seminiferous tubules in two experiments. LacZ staining of the recipient testes showed that the numbers of colonies generated from EpICD-GS and control GS cells were 210.4±8.4 and 298.1±34.1/10^5^ transplanted cells, respectively. The value for EpICD-GS cells was not significantly different from control value (Fig. 4G and H). Histological analysis of the recipient testes showed normal spermatogenesis (Fig. 4I). Thus, overexpression of EpICD did not appear to change SSC activity.

**Figure 5:**
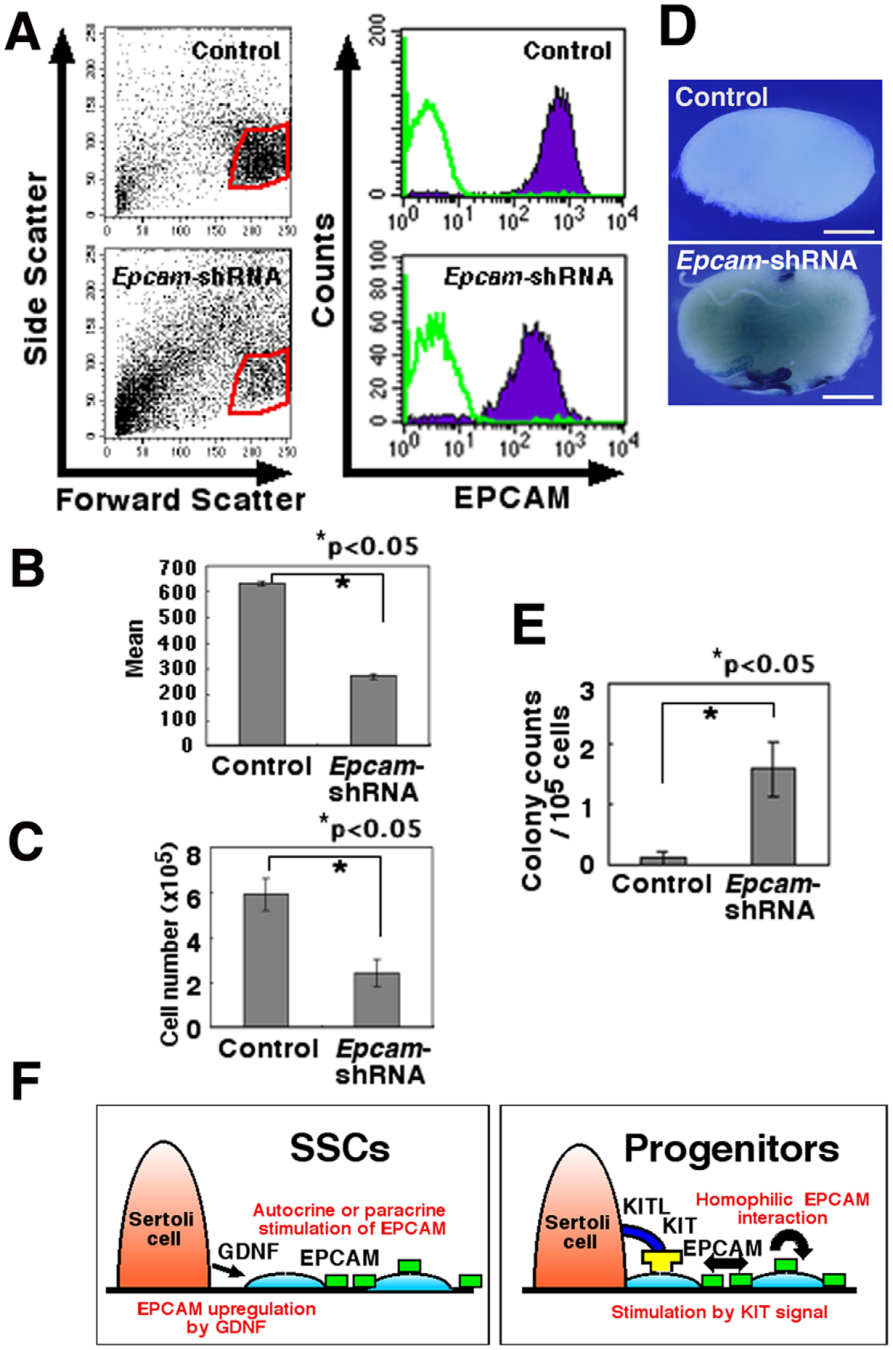
*Epcam* KD in ROSA GS cells by shRNA. (A) Flow cytometric profile of ROSA GS cells 2 days after transduction with *Epcam* KD vector. Green lines indicate controls. (B) Expression of EPCAM represented by the mean fluorescence intensity. Data are expressed as mean fluorescence intensity minus background autofluorescence of cells stained with the control secondary antibody. (C) Reduced recovery of ROSA GS cells after transduction with shRNA against *Epcam*. GS cells (8×10^5^) on MEFs were infected with the lentivirus and recovered 2 days later. Results of three experiments are shown. (D) Macroscopic appearance of the recipient testes transplanted with *Epcam* KD GS cells. The same number of cells was transplanted at the same time. Blue tubules indicate germ cell colonies developed from donor SSCs. (E) Assessment of SSC activity by germ cell transplantation. ROSA GS cells were transplanted into the seminiferous tubules of W mice 3 days after transduction with shRNA against *Epcam*. (F) A model for EPCAM function during SSC differentiation. GDNF upregulates EPCAM expression on SSCs/progenitors. Proliferation/survival of progenitors may be stimulated by EPCAM expression on neighboring cells as well as by KITL on Sertoli cells. Stain: X-Gal (D). Bar  = 1 mm (D).

In the second set of experiments, we used shRNA to inhibit EPCAM expression. Transfection of ROSA GS cells with *Epcam* KD lentivirus vector significantly suppressed EPCAM expression within 2 days (Fig. 5A and B). EPCAM downregulation suppressed the proliferation/survival of GS cells (Fig. 5C). Only 30.4±7.6% of the input cells were recovered after *Epcam* KD treatment, whereas 73.8±8.8% of the input cells could be recovered after control shRNA treatment. These results indicate that EPCAM plays a role in the proliferation or survival of GS cells.

We next evaluated the SSC activity by transplanting the *Epcam* KD-transfected cells into the seminiferous tubules. Two days after infection, the cultured cells were transplanted into the testes of W mice. In two separate experiments, *Epcam* KD cells and control GS cells generated 1.6±0.5 and 0.1±0.1 colonies/10^5^ transplanted cells, respectively; the difference was statistically significant (Fig. 5D and E). Thus, the *Epcam* KD treatment increased the concentration of SSCs in GS cell cultures.

## Discussion

Although EPCAM has been considered as a homophilic cell adhesion molecule, a series of recent studies has shown that EPCAM, upon cleavage into small fragments, transmits proliferation signals [14], [27], [28]. The short intracellular domain EpICD binds to a scaffolding protein, four-and-a-half LIM domains protein 2, and is translocated into the nucleus, where it becomes part of a large nuclear complex containing CTNNB1 and LEF1, two components of the Wnt pathway. This causes upregulation of MYC and cyclins, thereby facilitating proliferation. Consistent with this, *Epcam* KD compromised proliferation of embryonic stem (ES) cells [29]. EPCAM is also closely related to the maintenance of the undifferentiated state; EPCAM was downregulated by leukemia inhibitory factor (LIF) withdrawal, and KD treatment led to extensive differentiation [29]. As exogenously expressed EPCAM could only partially compensate for the requirement of ES cells for LIF, EPCAM is considered to be essential, but not sufficient for maintenance of the ES cell phenotype. Similar observations have also been reported for human ES cells [30], [31]. However, little progress has been made on the analyses of EPCAM expression and its function in the germline.

In the present study, EPCAM expression changed dynamically during SSC differentiation. We originally hypothesized that given its strong expression on GS cells, EPCAM would be a useful antigen for selection of a pure spermatogonial population from the testis in order to initiate GS cell culture without contamination by testicular somatic cells. However, EPCAM-selected cells had limited clonogenic activity in vitro, and showed strong KIT expression, suggesting that they were more enriched for progenitor spermatogonia compared with CD9-selected cells. Double immunostaining and transplantation of fractionated CD9-selected cells revealed that CD9^+^EPCAM^low/-^ cells showed little KIT expression, and had significantly increased SSC activity. These results support the suggestion that EPCAM is gradually upregulated in SSCs as they differentiate into progenitor spermatogonia in vivo.

EPCAM upregulation during SSC differentiation was unexpected, because EPCAM has been considered a useful marker for SSCs, including those in rat and humans [11], [32], and is strongly expressed on mouse GS cells. In fact, EPCAM was reported to be the best marker for rat SSCs [11]. Another study in rats also demonstrated clonogenic activity of EPCAM-expressing gonocytes [33]. Although these previous results strongly suggested that EPCAM expression in SSCs is conserved across different species, our results in mouse cells showed that EPCAM is regulated in a more sophisticated manner, being most strongly expressed in progenitor spermatogonia. The mechanism of SSC commitment has been a major topic of recent SSC research, but the lack of appropriate cell surface makers has prevented detailed analyses. EPCAM appears to be a useful cell surface marker for fractionating the spermatogonial compartment in studies of SSC self-renewal and differentiation. Our results also underscore the importance of functional transplantation studies based on the quantitative assessment of cell surface marker expression levels. It will be interesting to learn whether similar EPCAM expression patterns are conserved during spermatogenesis in other animal species.

The regulation and function of EPCAM were analyzed using GS cells, a pure proliferating spermatogonial cell population. EPCAM was upregulated by GDNF, suggesting that strong EPCAM expression in GS cell cultures is attributable in part to continuous exposure to GDNF, which is necessary for the propagation of SSCs in vitro. In contrast, FGF2 showed no apparent effect on EPCAM expression, although it is also an indispensable cytokine for GS cell culture. In ES cells, EPCAM expression is upregulated by LIF. Thus, our results indicate that the regulation of EPCAM expression differs between ES cells and germline cells. In GS cell culture, LIF is useful for initiating cultures from gonocytes, but is dispensable for establishment of GS cells from spermatogonia. We also have not been able to observe a positive effect of LIF on GS cell maintenance [34]. It may be that EPCAM expression changes in accordance with the cytokine milieu of the testicular micronevironment.

Although we did not find a significant effect of EpICD overexpression, the downregulation of EpICD by *Epcam* KD treatment significantly suppressed the GS cell recovery. This suggested that EPCAM is involved in proliferation or survival of spermatogonia. Interestingly, transplantation of *Epcam* KD cells resulted in a relative enrichment of SSCs. Given the in vivo expression pattern, these results suggest that EPCAM plays an important functional role in progenitor cell compartment. At present, very little is known about how progenitor spermatogonia increase their numbers in vivo. KIT is one factor involved in spermatogonial proliferation/survival [20]. Although its inhibition by neutralizing antibody kills a large number of proliferating spermatogonia [20], the inhibition of KIT signaling did not interfere with GS cell proliferation [35]. Similarly, the addition of KITL (Steel factor) did not enhance GS cell proliferation. Therefore, KIT does not appear to be vital in GS cell proliferation. The present results suggest that EPCAM may be a good candidate for progenitor cell proliferation. Cell-to-cell contact has been identified as an initial trigger for EPCAM activation [27]; therefore, we speculate that upregulated EPCAM on the cell surface may stimulate the proliferation of neighboring spermatogonia by shedding extracellular domain of EPCAM, thereby creating a positive feedback loop on proliferation signal in an autocrine or paracrine fashion [14]. This provides an additional stimulus to KIT, the ligand of which is expressed on Sertoli cells [20]. The availability of two different stimuli in parallel may perhaps contribute to the marked expansion of spermatogonia progenitors during differentiation (Fig. 5F).

The fractionation of CD9-selected cells based on EPCAM expression significantly improved the SSC purification efficiency. Subfractionation of the CD9-selected cells resulted in more efficient selection and achieved 48.7-fold enrichment. Assuming that 10% of SSCs can colonize seminiferous tubules [5], the frequency of SSCs in the suspension was 1 in 115 cells. Hence, this method appears to be more efficient than the in vivo enrichment method using cryptorchid testes, in which 1 in 161 cells were SSCs [36]. The high SSC activity in CD9^+^EPCAM^low/-^ cell population was in agreement with stronger expression of several spermatogonia molecules implicated in SSC self-renewal, including *Nanos3*, *Bcl6b*, and *Etv5* [8], [22]. However, the expression level of *Nanos2*, which is thought to be expressed in the most undifferentiated spermatogonia [21], was relatively weak in the same population, possibly be due to its low expression level or the small population size.

Previous attempts to enrich SSCs were based on cryptorchid mouse models with only undifferentiated spermatogonia. Although SSCs have now been purified to 1 in 15 to 30 cells by sorting of cells from the cyptorchid testes, the preparation of cryptorchid testes requires at least 2 months to remove differentiating germ cells [36], and the technique may not be applicable to many animal species due to differences in anatomical structures. We also cannot exclude the possibility that SSCs in cryptorchid testes have different biological characteristics from those in wild-type controls. For example, a recent study showed that KIT-expressing progenitor spermatogonia from wild-type testes can generate SSCs [37]. This was in contrast to our previous study that showed the absence of KIT on SSCs collected from cryptorchid testes [6]. In the present study, SSC activity was enriched in the CD9^+^EPCAM^low/-^ cell population, which consisted predominantly of KIT^low/-^ cells. However, this population also contained some KIT^−^ cells. Although we recently reported that both KIT^−^ and KIT^+^ cell populations in GS cell culture showed comparable levels of SSC activity [35], only KIT^−^cells showed SSC activity after transplantation, which suggested that KIT expression on SSCs may change according to their environment. Therefore, it is important to establish methods to purify SSCs from wild-type testes, and introduction of KIT as an additional marker may not only reconcile these conflicting observations but also improve the purification efficiency.

Ideally, the identification of SSC-specific antigens will greatly advance our understanding of SSC biology, as the lack of such markers has limited our knowledge regarding the regulation of SSC self-renewal and differentiation. Although the morphological description of spermatogonia has been well established, little progress has been made in the functional analysis of this compartment. Our results suggest that EPCAM is a useful marker for characterizing the spermatogonial compartment, and our analyses suggest that it plays an important role in spermatogonial progenitor proliferation or survival. The future analysis of this molecule will not only contribute to an improved SSC purification strategy but also increase our knowledge of SSC commitment.

## Supporting Information

Table S1 PCR primers.
(DOC)
