# Supplementary material for: Dynamic Changes in EPCAM Expression during Spermatogonial Stem Cell Differentiation in the Mouse Testis
Source: PLoS One. 2011 Aug 15;6(8):e23663. doi: 10.1371/journal.pone.0023663 (PMC3156235; doi:10.1371/journal.pone.0023663)
Supplement: Table S1 — PCR primers. (DOC) [file pone.0023663.s001.doc]

Table S1 PCR primers

| Gene | Primer sequence  Forward | | | Reverse |
| --- | --- | --- | --- | --- |
| **RT-PCR**  *Hprt*  *Pou5f1*  *Neurog3*  *Stra8*  *Zbtb16*  *Ret*  *Taf4b*  **Real-time PCR**  *Hprt*  *Pou5f1*  *Neurog3*  *Nanos2*  *Nanos3*  *Bcl6b*  *Etv5*  *Ccnd1*  *Ccnd2*  *Ccnd3* | GCTGGTGAAAAGGACCTCT  TTTCCCTCTGTTCCCGTCAC  GCCTCATTGGAGGAATTCC  AACGGTATCTCAACTTTTACAAGCA  GAGACACACAGACAGACCCATACT  CAGCGGTGTCTCCATCCAGTA  AGCCTAACAGCCACCAAACC  GCTGGTGAAAAGGACCTCT  TTTCCCTCTGTTCCCGTCAC  GCCTCATTGGAGGAATTCC  CCATATGCAACTTCTGCAAGC  CTTCTGTCTACTGCTACACCACC  ACTCCTCCGACGTGCTTAGC  TCTCGATCAGAGGACTGTCG  GTTCATTTCCAACCCACCC  GATCACCCACACTGATGTGG  ATGTCACAGCCATTCACCTG | | | CACAGGACTAGAACACCTGC  TGATCAACAGCATCACTGAGC  AGATGCTTGAGAGCCTCCAC  ATTTCTCCTCTGGATTTTCTGAGTT  CACACATAACACAGGTAGAGGTACG  CCCCTCGCTCGTGTCCTCCAA  TGAATTCTCAGCGGCATG  CACAGGACTAGAACACCTGC  TGATCAACAGCATCACTGAGC  AGATGCTTGAGAGCCTCCAC  TGAGTGTATGAGCCTGGTCG  TTGGAACCTGCATAGACACC  GGCCCCGGAAAATTGAATAG  CTTCAGCTAACCAAGCCTCC  CTCAGATGTCCACATCTCGC  ATGACGAACACGCCTCTCTC  CTGGTTGAGTGGGAAGGAAG |
|  |  |  |  | |
